# Reappraising Ischemic Heart Disease in Women

**DOI:** 10.31083/j.rcm2404118

**Published:** 2023-04-18

**Authors:** Jaclyn Carberry, Louise Aubiniere-Robb, Anna Kamdar, Harriet Lomholt-Welch, Colin Berry

**Affiliations:** ^1^British Heart Foundation Glasgow Cardiovascular Research Centre, University of Glasgow, G12 8QQ Glasgow, Scotland, UK; ^2^The West of Scotland Heart and Lung Centre, NHS Golden Jubilee, G81 4DY Glasgow, Scotland, UK

**Keywords:** ischemic heart disease, microvascular disease, sex, angina, pathophysiology, prognosis

## Abstract

Despite advances in the management of ischemic heart disease worldwide, mortality in women remains disproportionally high in comparison to men, particularly in women under the age of 55. The greater prevalence of ischemia with non-obstructive coronary arteries (INOCA) in women has been highlighted as a potential cause of this disparity. Moreover, current guideline recommendations for computed tomography coronary angiography (CTCA) as the first line of investigation for stable chest pain may further amplify this inequality. Traditional cardiovascular risk factors carry greater influence in women than men in the development of ischemic heart disease. Despite this, women have been consistently under-represented in large-scale clinical trials. Chest pain in women is more likely to be overlooked due to the higher likelihood of atypical presentation and normal anatomical imaging, despite persistent symptoms and decreased quality of life indicators. Accordingly, we call into question a CTCA-first approach in clinical guidelines; instead, we favor a personalized, patient first approach. Due to the misdiagnosis of ischemic heart disease in women, a large proportion are denied access to preventative therapy. This is especially true of women with INOCA, for which there is a critical lack of specific guidelines and rigorous evidence-based therapies. Ongoing clinical trials aim to identify potential management options that may benefit those with INOCA, bringing the field closer to eliminating sex-related disparities in the diagnosis, management and prognosis of ischemic heart disease.

## 1. Introduction

**Fig. 1.**
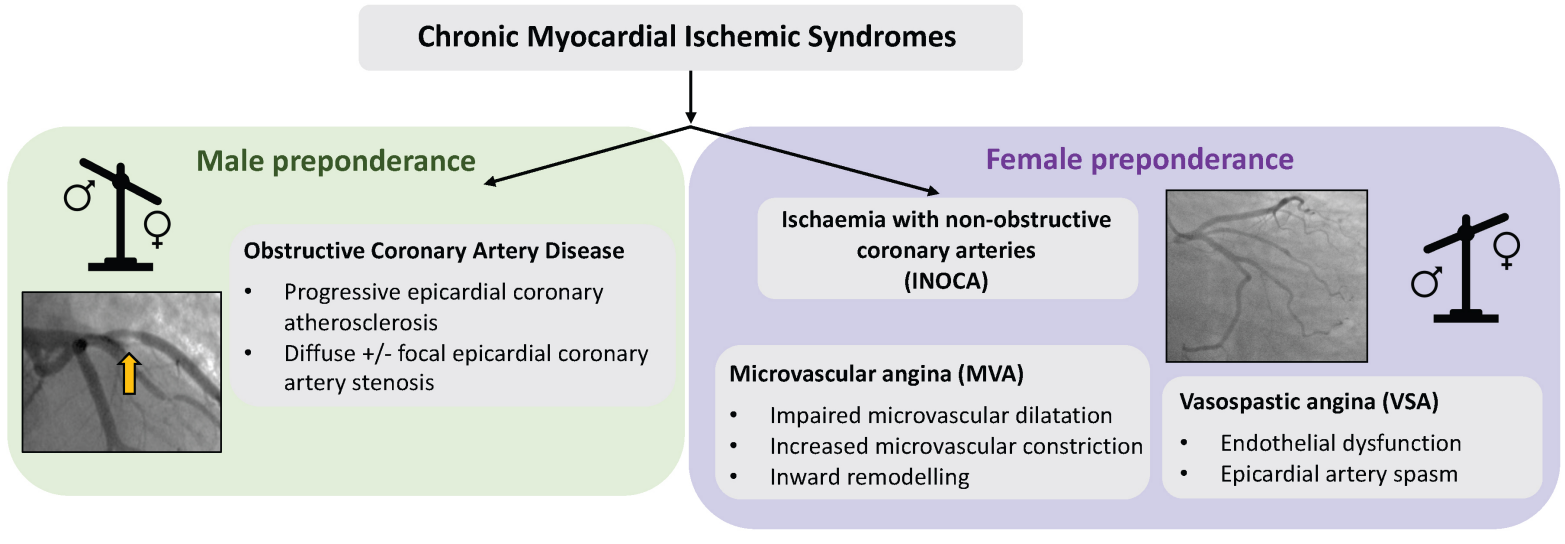
**Subtypes and sex preponderance in chronic myocardial ischemic syndromes**. Men are more likely to be diagnosed with obstructive coronary artery disease and women are more likely to suffer from INOCA. Acute myocardial ischemic syndromes display the same sex differences.

Ischemic heart disease remains a leading cause of death in both men and women, and in 2020 more women lost their lives to ischemic heart disease than to breast cancer [1]. Despite overall declining mortality in previous decades, mortality has declined to a lesser degree in women, particularly those under the age of 55 [2]. The persistently high death rate in younger women from ischemic heart disease merits scrutiny, and is even more concerning given that pre-menopausal women are naturally protected from cardiovascular events [3]. Where men are more likely to be diagnosed with obstructive coronary artery disease, women are more likely to suffer from angina or ischemia with non-obstructive coronary arteries (INOCA), conditions which are not benign (Fig. 1) [4]. Despite women having lower atherosclerotic plaque burden than men, they have a higher symptom burden of angina, poorer quality of life, increased hospitalization rates and a higher incidence of death [5, 6]. Women with INOCA have worse outcomes than men [7]. Moreover, women are more likely to undergo repeat coronary angiography for atypical symptoms, and are three times more likely to experience major adverse cardiovascular events within the first year of having an angiogram [8].

Given the historical prioritization of research funding on coronary artery disease, there is a critical deficit of evidence for the diagnosis and treatment of INOCA [2]. Contemporary practice guidelines prioritize anatomical imaging as a first-line approach for the investigation of suspected coronary artery disease, which risks falsely reassuring negative diagnoses in patients with INOCA, the majority of whom are women.

In this review, we will outline sex differences in the development and prognosis of ischemic heart disease, the problems with current guideline recommendations, evidence-based treatments for ischemic heart disease in women and strategies for resolving sex disparities in the research and clinical landscape.

## 2. Defining Ischemia with Non-Obstructive Coronary Artery Disease (INOCA)

Historically, ‘syndrome X’ was a term used to describe a group of patients with anginal chest pain of uncertain etiology. This term had uncertain meaning and partly reflected therapeutic nihilism. Since most affected individuals were female, this term promulgated sex-related disparities in healthcare. This paradigm is evolving, and the term ‘syndrome X’ has been replaced with INOCA – a term which better reflects the abnormalities the condition, enhancing understanding and the potential for evidence-based targeted therapies. Advances in diagnostic techniques, enhanced access, clinical evidence and patient and public involvement are beginning to move the field forward [2].

Ischemic heart disease is a unifying term, reflecting the end-organ problem of myocardial ischemic syndromes which may be acute or chronic. Second order, major subgroups leading to chronic myocardial ischemic syndromes include obstructive coronary artery disease or INOCA reflecting endotypes defined by distinct disease mechanisms (Fig. 1). INOCA endotypes include microvascular angina, vasospastic angina and coronary endothelial dysfunction. Microvascular angina may be functional and/or structural. Functional microvascular angina is caused by impaired small vessel vasodilatation and/or microvascular spasm leading to myocardial ischemia. Structural microvascular angina is due to small vessel remodelling and/or interstitial changes limiting blood flow to the myocardium on demand. Vasospastic angina is caused by spasm of the epicardial conduit coronary artery [2]. As highlighted in the Stratified Medical Therapy Using Invasive Coronary Function Testing in Angina (CorMicA) trial, these endotypes may co-exist [9]. They may also occur in patients with obstructive coronary artery disease and may underlie angina post-percutaneous coronary intervention.

## 3. Women have Different Risk Factors for Ischemic Heart Disease than Men

The risk factor profile and the impact of risk factors for ischemic heart disease differ between men and women (Table 1). Traditional risk factors, such as smoking, diabetes mellitus, dyslipidemia, lack of physical activity, hypertension, obesity, and ageing, affect both men and women. However, many of these risk factors portend a higher risk of ischemic heart disease in women than in men [10]. Concurrent renal dysfunction, for example, has been associated with greater risk of adverse cardiovascular outcomes in women with angina [11, 12]. Perpetuating this issue, women are underrepresented in clinical trials of risk reduction for cardiovascular disease prevention, relative to the prevalence of disease in the population [13]. There is an unmet need for proven preventative therapies which are adequately evidenced in both men and women, or which are sex specific.

**Table 1.** **Common and sex-specific/emerging risk factors for ischemic heart disease in women**.

Common	Sex-specific and emerging
Smoking	Pregnancy
Diabetes mellitus	Gestational diabetes
Dyslipidemia	Pre-eclampsia
Sedentary lifestyle	Menopause (including premature menopause)
Hypertension	Autoimmune conditions (e.g., rheumatoid arthritis, systemic lupus erythematosus)
Obesity	Breast cancer
Age	Mental stress
Renal dysfunction	

There are several non-traditional risk factors unique to women for the development of ischemic heart disease. These include pregnancy and pregnancy-related complications (e.g., gestational diabetes and pre-eclampsia), menopause, and autoimmune rheumatological conditions with higher female: male prevalence, such as rheumatoid arthritis and systemic lupus erythematosus [10, 14]. In a large population-based study, 61% of those with autoimmune disease were women, and the risk of cardiovascular disease in those with autoimmune disease was approximately 1.5× that of those without an autoimmune disease [15]. Additionally, women who have survived breast cancer are at increased risk of ischemic heart disease, partly due to therapies such as chest wall radiation [16]. A further non-traditional risk factor affecting more women than men is mental stress. In the Stabilization of Atherosclerotic Plaque by Initiation of Darapladib Therapy (STABILITY) trial, women with a history of coronary artery disease had better clinical outcomes than men. However, when the association was adjusted for the frequency of self-reported depressive symptoms, the cardiovascular risk was equalized [17].

The role of estrogen has been investigated as a possible explanation for sex differences in presentation of ischemic heart disease. The drop in estrogen that occurs post-menopause results in specific conditions (e.g., the redistribution of subcutaneous fat to the viscera) which are hypothesized to be a contributing factor to the development of coronary microvascular disease in women, and has therefore been investigated as a potential therapeutic target [18]. Two large randomized controlled trials, the Women’s Health Initiative (WHI) and the Heart and Estrogen/progestin Replacement Study (HERS), showed no evidence of benefit for primary or secondary prevention with menopause hormone therapy [19, 20]. If commenced within the optimal timing window (<60 years of age or within 10 years since the last menstrual period), menopause hormone therapy can reduce cardiovascular morbidity and mortality, although it is contraindicated for the sole purpose of prevention in women at high risk of cardiovascular disease [21].

## 4. Diagnosing Ischemic Heart Disease in Women

Women are less likely to have investigations performed for chest pain and, accordingly, are at risk of underdiagnosis, undertreatment, and poorer prognosis [22, 23]. The definition of “typical” angina less often applies to women than it does to men, which is a likely contributing factor to these worrying statistics. Indeed, in the Outcomes of Anatomical versus Functional Testing for Coronary Artery Disease (PROMISE) trial, physician characterization of chest pain was more likely to be nonanginal in women, even though the women in the trial had more cardiovascular risk factors than the men [24]. Women experience a more diverse cluster of symptoms than men, such as dyspnoea, palpitations, diaphoresis, or fatigue, and are more likely to have non-exertional symptoms [25, 26]. However, women are just as likely to describe chest pain as men, and most patients who present to the emergency department with chest pain are women [22].

### 4.1 Investigating Stable Chest Pain Using a Computed Tomography Coronary Angiography (CTCA)-First Approach

Most patients with suspected ischemic heart disease do not have obstructive coronary artery disease [27, 28], indicating that the majority (approximately 4 in 5 individuals) have an alternative cause, notably, INOCA. Approximately three-quarters of patients with INOCA are women [29, 30, 31]. An anatomical testing approach could result in many women with INOCA being falsely reassured and discharged, despite having a treatable underlying etiology. This was clearly reflected in a prespecified subanalysis of the Calcium Imaging and Selective CT Angiography in Comparison to Functional Testing for Suspected Coronary Artery Disease (CRESCENT) trial, where CTCA decreased time to diagnosis to a greater extent in women than in men [32] (Table 2, Ref. [24, 27, 28, 32, 33, 34, 35, 36, 37]). Further, a large meta-analysis of prospective diagnostic accuracy studies demonstrated that the diagnostic performance of CTCA was slightly lower in women than in men [33] (Table 2).

**Table 2.** **Large-scale CTCA trials performed with key sex differences highlighted**.

Study and year	Study groups	Key inclusion criteria	Number of participants (% female)	Primary outcome and results	Key sex differences
SCOTHEART 2015 [28]	CTCA + standard care vs standard care	Referred to hospital by primary-care physician with suspected stable angina due to coronary artery disease	4146 (43.9)	Diagnosis reclassified more often in the CTCA group	CTCA resulted in more women being reclassified as not having coronary artery disease
		Age 18–75 years old		23% vs 1%; *p *< 0.001	Absolute risk difference 5.7 (2.7–8.7); *p *< 0.001 [34]
PROMISE 2016 [27]	CTCA vs functional testing	Symptomatic outpatients without coronary artery disease and physician belief that noninvasive/nonurgent imaging required for suspected coronary artery disease	10,003 (52.7)	Composite of death from any cause, myocardial infarction or hospitalization for unstable angina occurred in 3.3% of CTCA vs 3.0% of functional testing	Women more likely to be sent for imaging stress tests than non-imaging tests
		Age 45–54 male, 50–64 female		HR 1.04 (95% CI 0.83–1.29); *p* = 0.075	OR 1.21 (1.01–1.44); *p* = 0.043 [24]
		≥1 cardiac risk factor			
CRESCENT 2016 [35]	CTCA vs functional testing	Stable chest pain or angina equivalent potentially caused by coronary artery disease	350 (55.3)	Fewer participants had chest pain at 1 year follow-up in the CTCA group	No sex interaction observed for the primary outcome of angina at 1 year or quality of life (all *p *≥ 0.097)
		≥18 years old		19% vs 25%; *p* = 0.012	CTCA decreased diagnosis time in women to a greater extent than men (*p* = 0.012) [32]
				No differences in quality of life between groups (*p* = 0.759)	
CAD-Man 2016 [36]	CTCA vs coronary angiography	Patients presenting with atypical angina pectoris with suspected coronary artery disease and coronary intervention planned	329 (50.4)	No difference in major procedure complications	*None reported*
		Age ≥30 years old		0.6% CTCA vs 0% coronary angiography (*p * = 1.00)	
COME-CCT 2019 [33] (Prospectively designed meta-analysis)	CTCA vs coronary angiography	Patients who have undergone both CTCA and coronary angiography indicated due to stable chest pain	5332 (34.9)	At a pre-test probability of 7%, positive predictive value of CTCA was 50.9% (43.3%–57.7%), negative predictive value 97.8% (96.4%–98.7%).	Diagnostic performance of CTCA was slightly lower in women than in men
		Coronary artery disease with diameter stenosis of ≥50%		At pre-test probability of 67%, positive predictive value 82.7% (78.3%–86.2%), negative predictive value 85.0% (80.2%–88.9%)	Area under the curve 0.874 (0.858–0.890) vs 0.907 (0.897–0.916); *p *< 0.001
DISCHARGE 2022 [37]	CTCA vs coronary angiography	Referred for invasive coronary angiogram with stable angina and intermediate likelihood of obstructive disease	3561 (56.3)	Composite of cardiovascular death, non-fatal myocardial infarction or nonfatal stroke occurred in 2.1% in CTCA vs 3.0% in coronary angiography group	*None reported*
		Age ≥30 years old		HR 0.26 (0.13–0.55); *p* = 0.10	

Abbreviations: CI, confidence interval; CTCA, computed tomography coronary angiogram; HR, hazard ratio; OR, odds ratio.

Despite this, the 2016 National Institute for Health and Care Excellent (NICE)-95 clinical guideline for the investigation of chest pain recommends a CTCA-first approach [38]. This guideline still includes a 2010 recommendation for the consideration of “Syndrome X” in patients without flow-limiting disease, perpetuating unhelpful, outdated and sex-bias terminology [38]. NICE-95 and Scottish Intercollegiate Guidelines Network (SIGN)-151 both fail to consider INOCA within the primary test strategy. Stakeholder organizations have recognized this as a potential societal problem [39]. Positioning CTCA as the primary diagnostic strategy in patients with angina will systematically favor a positive diagnosis in individuals with obstructive coronary disease, who are the minority of individuals and are mostly male.

Landmark CTCA trials did not show any benefit in long-term cardiovascular outcomes using a CTCA-first approach (Table 2). Further, a CTCA-guided approach to diagnosis and treatment attenuates improvements in quality of life and symptom burden [40]. A misdiagnosis of non-cardiac chest pain following a negative CTCA is likely to leave patients with higher levels of anxiety and confusion about their symptoms, and lead to a host of unnecessary investigations for a non-cardiac cause. The British Heart Foundation Coronary Microvascular Function and CT Coronary Angiogram (CorCTCA) study will highlight the scale of the problem by assessing the prevalence of INOCA amongst patients with no obstructive coronary artery disease on CTCA, and will also assess the effect of a stratified treatment approach on wellbeing [41].

The 2019 European Society of Cardiology (ESC) guidelines stratify recommendations for the investigation of ischemic heart disease based on an initial assessment of risk using the updated Diamond-Forrester risk score. Patients at lower risk of coronary artery disease are recommended CTCA, and those at higher risk are recommended non-invasive functional imaging [42]. The updated Diamond-Forrester risk score was developed in a high-risk population (67% male, with around two-thirds having angiographic evidence of obstructive coronary artery disease), and is more likely to reclassify women into a lower-risk category than men [43]. Women are more likely to have a lower pre-test probability for coronary artery disease, and are therefore more likely to be investigated with CTCA according to the European guidelines, presenting the same disadvantages to women as the NICE and SIGN guidelines [25]. The prediction of coronary artery disease is improved when incorporating female-specific risk factors into risk scores, however, this is not incorporated in clinical practice [44].

### 4.2 Functional Testing for Ischemic Heart Disease

Functional testing for ischemic heart disease includes invasive and non-invasive investigations. Invasive coronary function testing includes pressure wire and thermodilution assessment of coronary flow reserve (CFR) and index of microvascular resistance to test for microvascular dysfunction, and acetylcholine provocation testing for vasospasm [9]. Non-invasive testing includes nuclear myocardial scintigraphy (MPS), stress echo, stress cardiac magnetic resonance imaging (CMR) and exercise treadmill testing.

**Fig. 2.**
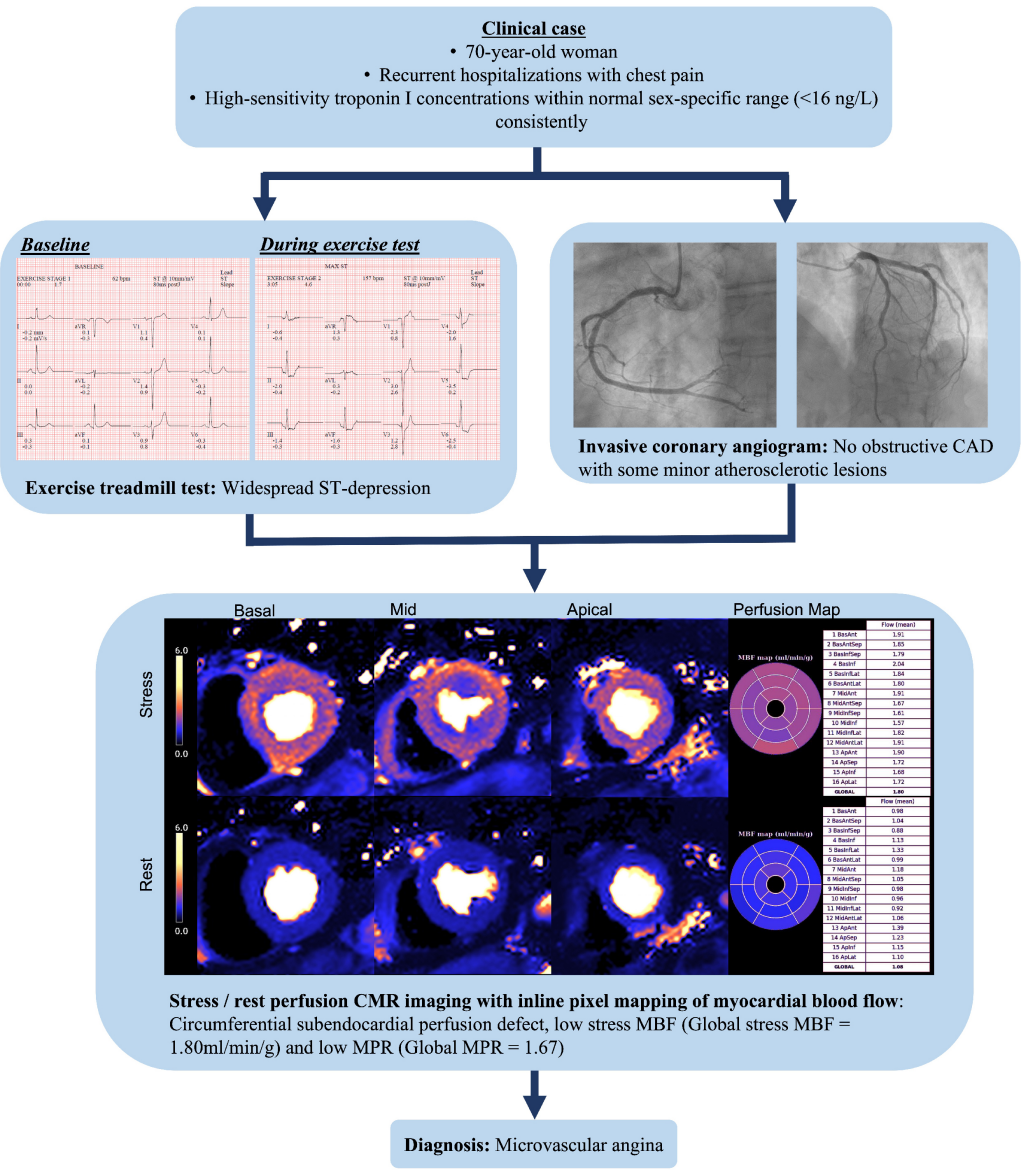
**Clinical case of ischemia with non-Obstructive coronary artery Disease (INOCA)**. A 70-year-old woman with recurrent hospitalizations with chest pain. High-sensitivity troponin I concentrations measured within the normal sex-specific range (<16 ng/L). The exercise treadmill test was strongly positive for ischemia with widespread horizontal ST-segment depression. The invasive coronary angiogram showed minor atherosclerotic plaque only. CMR stress and rest imaging revealed a circumferential subendocardial perfusion defect, low stress MBF (Global stress MBF = 1.80 mL/min/g) and low MPR (Global MPR = 1.67). The final diagnosis was microvascular angina. Abbreviations: CAD, coronary artery disease; CMR, cardiac magnetic resonance imaging; MBF, myocardial blood flow; MPR, myocardial perfusion reserve. (Acknowledgement to Dr C Bradley, Dr P Kellman and Dr H Xue, National Institutes of Health).

MPS is the most specific of the non-invasive options for myocardial ischemia [45]. However, in women, accuracy is lower due to smaller heart size and higher left ventricular ejection fraction, and is less frequently the first investigation of choice in women due to increased radiation exposure [46]. CMR has the potential to be a preferred non-invasive functional imaging option for patients with suspected INOCA. CMR offers high-resolution and multiparametric imaging techniques, without the risks associated with radiation exposure. On the other hand, CMR imaging is expensive and access to services can be limited. The ongoing Coronary Microvascular Angina Cardiac Magnetic Resonance Imaging (CorCMR) study will determine whether CMR-guided therapy in patients with angina without obstructive coronary disease will result in improved symptom control and well-being (NCT04805814) (Fig. 2).

## 5. Management of Ischemic Heart Disease in Women According to the Mechanism

Management of ischemic heart disease aims to reduce ischemia, alleviate symptoms, and improve quality of life. Gender disparity in the management of ischemic heart disease exists for several reasons. Firstly, the use of guideline-directed medical therapy for ischemic heart disease is suboptimal in women [47]. Secondly, and arguably more importantly, sex-stratified guidelines on the management of ischemic heart disease are lacking; current practice recommendations are primarily based on studies in men [48]. The underlying pathophysiological mechanism which are unique to women may respond differently to treatment compared with that in men, highlighting the need for sex-specific research and treatment guidelines [49].

Initiation of treatment for ischemic heart disease is more commonly delayed in women compared to men, leading to reduced prescribing of guideline-recommended medications and late onward referral in patients with refractory or under-treated symptoms [47]. Fewer women with ischemic heart disease are treated with statins despite research showing similarly improved cardiovascular outcomes with lipid-lowering therapy for primary and secondary prevention [50].

Women with stable angina are not only less likely to undergo invasive coronary angiography compared with men, but are also less likely to receive appropriate revascularization therapy [51]. Coronary artery bypass grafting (CABG) is a treatment option in both male and female patients with significant obstruction of the left main stem or with triple-vessel disease. However, women who undergo CABG experience less symptomatic relief than men. Some investigators have attributed poorer CABG outcomes in women to smaller vessel diameter leading to higher rates of incomplete revascularization [52].

Contemporary guidelines for the management of angina are not targeted at the underlying mechanism. This is mainly because validated data on the optimal pharmacotherapeutic management of INOCA is limited [53]. This may be an important factor in explaining why treatment, symptom control and patient satisfaction in women is suboptimal. Incorporating INOCA as an independent diagnosis into practice guidelines will facilitate more favorable outcomes in women and decreased gender bias. The CorMicA trial provided evidence that a personalized therapeutic approach in patients with INOCA improves symptoms and quality of life relative to the current standard of care [54]. The Coronary Microvascular Function and Cardiovascular Risk Factors in Women With Angina Pectoris and No Obstructive Coronary Artery Disease (iPOWER) study demonstrated that weight reduction and risk factor optimization in women with coronary microvascular dysfunction in the absence of flow-limiting epicardial disease was associated with a significant reduction of angina severity, although this did not improve microvascular function [55]. These studies underpin the importance of coronary microvascular function testing, particularly in women, to optimize the treatment strategy to a specific diagnosis instead of using a generic “one size fits all” approach in all patients with angina.

## 6. Clinical Strategies for Eliminating Sex-Related Disparities in Ischemic Heart Disease

Eliminating sex-related bias starts with identifying the existing knowledge gaps in ischemic heart disease. One vital issue is the underrepresentation of women in cardiovascular trials [56]. Recruitment bias and lack of female participation has contributed to the paucity of sex-specific data on ischemic heart disease. Women represent around 30% of coronary artery disease trial populations, whilst representing 45% of the real-world population. Barriers to trial enrolment include reproductive stage, inclusion criteria that do not account for sex differences in cardiac biomarkers, and lack of gender diversity amongst trial investigators and committee members [56]. Another important barrier is social inequality which disproportionally affects women compared to men [57]. Currently there is no guideline-approved framework tackling gender bias in the provision of healthcare that has demonstrated significant benefit in women with cardiovascular disease [58].

Studies which outline potential therapeutic strategies to optimize management of ischemic heart disease focus on the pathophysiological subtype of angina. The Women’s Ischemia Syndrome Evaluation (WISE) demonstrated that angiotensin-converting enzyme (ACE) inhibitors reduce microvascular dysfunction and angina severity in women with INOCA. ACE inhibitors improve CFR in women without angiographic coronary artery disease and a low CFR at baseline [59]. There is evidence to suggest that statin/ACE inhibitor combination therapy may be superior to ACE inhibitors alone for improving microcirculatory function and symptom alleviation in patients with non-obstructive coronary artery disease [60]. Future work should close the evidence gap and eliminate sex-related disparities in the diagnosis and treatment of chronic coronary syndromes. Whilst the CorMicA trial is not sex-specific it sheds light on optimized treatment strategies in women, in whom INOCA is more prevalent. Several trials are ongoing, one of which is recruiting exclusively women (Table 3). The Women’s IschemiA Trial to Reduce Events In Non-ObstRuctive Coronary Artery Disease (WARRIOR) is a multicenter, prospective, randomized, blinded outcome evaluation studying the efficacy of intensive medical therapy (statin, ACE inhibitor plus aspirin) compared to standard care. The primary endpoint is first occurrence of major adverse cardiovascular events. Secondary endpoints include symptom severity, quality of life and healthcare resource utilization [61]. This promising clinical trial will guide future best practices by providing the necessary evidence to support the implementation of sex-stratified guidelines on ischemic heart disease.

**Table 3.** ** Emerging therapies for INOCA**.

Study	Clinical trial identifier	Inclusion criteria	n	Intervention	Primary outcome	Current progress
PRIZE	NCT04097314	Microvascular angina	356	Zibotentan 10 mg daily	Change in exercise treadmill test time	Ongoing. Expected completion November 2022
		Age ≥18 years old				
TIC-2	ACTRN12616000388415	Coronary slow flow in absence of obstructive coronary artery disease	35	Ticagrelor 90 mg twice daily for 4 weeks	Change in angina symptom frequency	Stopped early due to resource constraints
		Angina symptoms ≥3 times in the 2 weeks prior to enrolment				Data collected currently subject to analysis
		Age ≥18 years old				
WARRIOR	NCT03417388	Non-obstructive coronary artery disease	4422	High dose atorvastatin/rosuvastatin + lisinopril or losartan + lifestyle counselling ± aspirin vs primary prevention risk factor therapy	All-cause mortality during 3-year study period	Ongoing. Expected completion December 2023
		Female				
		Age 18–100 years old				
COSIMA	NCT04606459	Evidence of microvascular disease	144	Coronary sinus reducer	Change in Canadian Cardiovascular Society angina class ≥2 within 6-month study period	Ongoing. Expected completion October 2029
		Refractory angina				
		Canadian Cardiovascular Society angina class III–IV				
		Age ≥18 and ≤85 years old				
Rhodiola Rosea for coronary microvascular disease	NCT04218916	Typical angina pectoris with normal coronaries or a stenosis <20% and an anterior descending coronary flow reserve <2.0	114	0.56 g Rhodiola Rosea capsules three times a day	Change in coronary flow reserve after 1 year	Ongoing. Expected completion January 2023
		Age 40–75 years old				

INOCA, Ischemia with Non-Obstructive Coronary Artery Disease PRIZE, Precision Medicine With Zibotentan in Microvascular Angina; TIC-2, Ticagrelor in Coronary Microvascular Dysfunction 2 Trial; WARRIOR, Women’s Ischemia Trial to Reduce Events In Non-Obstructive Coronary Artery Disease; COSIMA, Coronary Sinus Reducer for the Treatment of Refractory Microvascular Angina.

## 7. Conclusions

Ischemic heart disease is the leading cause of mortality in both men and women worldwide. In recent decades, reductions in mortality have been largely observed only in men. The CTCA-first diagnostic approach risks the misclassification of INOCA as non-cardiac chest pain, preventing appropriate further investigation and management and disadvantaging mainly women. Treatment for women with ischemic heart disease remains suboptimal, and guidelines are largely based on research conducted in men with obstructive coronary artery disease. Improving the representation of women in large-scale cardiovascular outcome trials and INOCA-specific therapy trials are vital to improving the management of cardiovascular conditions affecting women. Greater awareness, further research, and updated guidelines are critical to reducing the sex-disparities in treatment, diagnosis and prognosis of ischemic heart disease.

## References

[1] British Heart Foundation (2022). Heart Statistics. https://www.bhf.org.uk/what-we-do/our-research/heart-statistics.

[2] Berry C (2017). Stable Coronary Syndromes: The Case for Consolidating the Nomenclature of Stable Ischemic Heart Disease. *Circulation*.

[3] Muka T, Oliver-Williams C, Kunutsor S, Laven JSE, Fauser BCJM, Chowdhury R (2016). Association of Age at Onset of Menopause and Time Since Onset of Menopause With Cardiovascular Outcomes, Intermediate Vascular Traits, and All-Cause Mortality: A Systematic Review and Meta-analysis. *JAMA Cardiology*.

[4] Hemingway H, Langenberg C, Damant J, Frost C, Pyörälä K, Barrett-Connor E (2008). Prevalence of angina in women versus men: a systematic review and meta-analysis of international variations across 31 countries. *Circulation*.

[5] Jespersen L, Hvelplund A, Abildstrøm SZ, Pedersen F, Galatius S, Madsen JK (2012). Stable angina pectoris with no obstructive coronary artery disease is associated with increased risks of major adverse cardiovascular events. *European Heart Journal*.

[6] Olson MB, Kelsey SF, Matthews K, Shaw LJ, Sharaf BL, Pohost GM (2003). Symptoms, myocardial ischaemia and quality of life in women: results from the NHLBI-sponsored WISE Study. *European Heart Journal*.

[7] Kenkre TS, Malhotra P, Johnson BD, Handberg EM, Thompson DV, Marroquin OC (2017). Ten-Year Mortality in the WISE Study (Women’s Ischemia Syndrome Evaluation). *Circulation. Cardiovascular Quality and Outcomes*.

[8] Sedlak TL, Lee M, Izadnegahdar M, Merz CNB, Gao M, Humphries KH (2013). Sex differences in clinical outcomes in patients with stable angina and no obstructive coronary artery disease. *American Heart Journal*.

[9] Ford TJ, Stanley B, Good R, Rocchiccioli P, McEntegart M, Watkins S (2018). Stratified Medical Therapy Using Invasive Coronary Function Testing in Angina: The CorMicA Trial. *Journal of the American College of Cardiology*.

[10] Isiadinso I, Shaw LJ (2016). Diagnosis and risk stratification of women with stable ischemic heart disease. *Journal of Nuclear Cardiology*.

[11] Chen R, Kumar S, Timmis A, Feder G, Yaqoob MM, Hemingway H (2006). Comparison of the relation between renal impairment, angiographic coronary artery disease, and long-term mortality in women versus men. *The American Journal of Cardiology*.

[12] Anavekar NS, McMurray JJV, Velazquez EJ, Solomon SD, Kober L, Rouleau JL (2004). Relation between renal dysfunction and cardiovascular outcomes after myocardial infarction. *The New England Journal of Medicine*.

[13] Melloni C, Berger JS, Wang TY, Gunes F, Stebbins A, Pieper KS (2010). Representation of women in randomized clinical trials of cardiovascular disease prevention. *Circulation: Cardiovascular Quality and Outcomes*.

[14] Honigberg MC, Zekavat SM, Aragam K, Klarin D, Bhatt DL, Scott NS (2019). Long-Term Cardiovascular Risk in Women With Hypertension During Pregnancy. *Journal of the American College of Cardiology*.

[15] Conrad N, Verbeke G, Molenberghs G, Goetschalckx L, Callender T, Cambridge G (2022). Autoimmune diseases and cardiovascular risk: a population-based study on 19 autoimmune diseases and 12 cardiovascular diseases in 22 million individuals in the UK. *Lancet*.

[16] Darby SC, Ewertz M, McGale P, Bennet AM, Blom-Goldman U, Brønnum D (2013). Risk of ischemic heart disease in women after radiotherapy for breast cancer. *The New England Journal of Medicine*.

[17] Guimarães PO, Granger CB, Stebbins A, Chiswell K, Held C, Hochman JS (2017). Sex Differences in Clinical Characteristics, Psychosocial Factors, and Outcomes Among Patients With Stable Coronary Heart Disease: Insights from the STABILITY (Stabilization of Atherosclerotic Plaque by Initiation of Darapladib Therapy) Trial. *Journal of the American Heart Association*.

[18] Willemars MMA, Nabben M, Verdonschot JAJ, Hoes MF (2022). Evaluation of the Interaction of Sex Hormones and Cardiovascular Function and Health. *Current Heart Failure Reports*.

[19] Anderson GL, Limacher M, Assaf AR, Bassford T, Beresford SAA, Black H (2004). Effects of conjugated equine estrogen in postmenopausal women with hysterectomy: the Women’s Health Initiative randomized controlled trial. *The Journal of the American Medical Association*.

[20] Hulley S, Grady D, Bush T, Furberg C, Herrington D, Riggs B (1998). Randomized trial of estrogen plus progestin for secondary prevention of coronary heart disease in postmenopausal women. Heart and Estrogen/progestin Replacement Study (HERS) Research Group. *The Journal of the American Medical Association*.

[21] Hodis HN, Mack WJ (2022). Menopausal Hormone Replacement Therapy and Reduction of All-Cause Mortality and Cardiovascular Disease: It Is About Time and Timing. *Cancer Journal*.

[22] Gulati M, Levy PD, Mukherjee D, Amsterdam E, Bhatt DL, Birtcher KK (2021). 2021 AHA/ACC/ASE/CHEST/SAEM/SCCT/SCMR Guideline for the Evaluation and Diagnosis of Chest Pain: A Report of the American College of Cardiology/American Heart Association Joint Committee on Clinical Practice Guidelines. *Journal of the American College of Cardiology*.

[23] Jordan KP, Rathod-Mistry T, Bailey J, Chen Y, Clarson L, Denaxas S (2022). Long-Term Cardiovascular Risk and Management of Patients Recorded in Primary Care With Unattributed Chest Pain: An Electronic Health Record Study. *Journal of the American Heart Association*.

[24] Hemal K, Pagidipati NJ, Coles A, Dolor RJ, Mark DB, Pellikka PA (2016). Sex Differences in Demographics, Risk Factors, Presentation, and Noninvasive Testing in Stable Outpatients With Suspected Coronary Artery Disease: Insights From the PROMISE Trial. *JACC: Cardiovascular Imaging*.

[25] Shaw LJ, Bairey Merz CN, Pepine CJ, Reis SE, Bittner V, Kelsey SF (2006). Insights from the NHLBI-Sponsored Women’s Ischemia Syndrome Evaluation (WISE) Study: Part I: gender differences in traditional and novel risk factors, symptom evaluation, and gender-optimized diagnostic strategies. *Journal of the American College of Cardiology*.

[26] Vitola B, Trusinskis K, Mintale I, Kalnina M, Erglis A (2022). Coronary Artery Disease in Women: Lessons Learned from Single-Center SPECT Registry and Future Directions for INOCA Patients. *Medicina*.

[27] Douglas PS, Hoffmann U, Patel MR, Mark DB, Al-Khalidi HR, Cavanaugh B (2015). Outcomes of anatomical versus functional testing for coronary artery disease. *The New England Journal of Medicine*.

[28] SCOT-HEART investigators (2015). CT coronary angiography in patients with suspected angina due to coronary heart disease (SCOT-HEART): an open-label, parallel-group, multicentre trial. *Lancet*.

[29] Ford TJ, Yii E, Sidik N, Good R, Rocchiccioli P, McEntegart M (2019). Ischemia and No Obstructive Coronary Artery Disease: Prevalence and Correlates of Coronary Vasomotion Disorders. *Circulation. Cardiovascular Interventions*.

[30] Reynolds HR, Bairey Merz CN, Berry C, Samuel R, Saw J, Smilowitz NR (2022). Coronary Arterial Function and Disease in Women With No Obstructive Coronary Arteries. *Circulation Research*.

[31] Aribas E, Roeters van Lennep JE, Elias-Smale SE, Piek JJ, Roos M, Ahmadizar F (2022). Prevalence of microvascular angina among patients with stable symptoms in the absence of obstructive coronary artery disease: a systematic review. *Cardiovascular Research*.

[32] Lubbers M, Coenen A, Bruning T, Galema T, Akkerhuis J, Krenning B (2017). Sex Differences in the Performance of Cardiac Computed Tomography Compared With Functional Testing in Evaluating Stable Chest Pain: Subanalysis of the Multicenter, Randomized CRESCENT Trial (Calcium Imaging and Selective CT Angiography in Comparison to Functional Testing for Suspected Coronary Artery Disease). *Circulation: Cardiovascular Imaging*.

[33] Haase R, Schlattmann P, Gueret P, Andreini D, Pontone G, Alkadhi H (2019). Diagnosis of obstructive coronary artery disease using computed tomography angiography in patients with stable chest pain depending on clinical probability and in clinically important subgroups: meta-analysis of individual patient data. *British Medical Journal*.

[34] Mangion K, Adamson PD, Williams MC, Hunter A, Pawade T, Shah ASV (2020). Sex associations and computed tomography coronary angiography-guided management in patients with stable chest pain. *European Heart Journal*.

[35] Lubbers M, Dedic A, Coenen A, Galema T, Akkerhuis J, Bruning T (2016). Calcium imaging and selective computed tomography angiography in comparison to functional testing for suspected coronary artery disease: the multicentre, randomized CRESCENT trial. *European Heart Journal*.

[36] Dewey M, Rief M, Martus P, Kendziora B, Feger S, Dreger H (2016). Evaluation of computed tomography in patients with atypical angina or chest pain clinically referred for invasive coronary angiography: randomised controlled trial. *British Medical Journal*.

[37] Maurovich-Horvat P, Bosserdt M, Kofoed KF, Rieckmann N, Benedek T, Donnelly P (2022). CT or Invasive Coronary Angiography in Stable Chest Pain. *The New England Journal of Medicine*.

[38] National Institute for Health and Care Excellence (2016). Recent-onset chest pain of suspected cardiac origin: assessment and diagnosis. https://www.nice.org.uk/guidance/cg95/resources/recentonset-chest-pain-of-suspected-cardiac-origin-assessment-and-diagnosis-pdf-975751034821.

[39] Bakker J (2019). Heart attack gender gap is costing women’s lives. https://www.bhf.org.uk/what-we-do/news-from-the-bhf/news-archive/2019/september/heart-attack-gender-gap-is-costing-womens-lives.

[40] Williams MC, Hunter A, Shah A, Assi V, Lewis S, Mangion K (2017). Symptoms and quality of life in patients with suspected angina undergoing CT coronary angiography: a randomised controlled trial. *Heart*.

[41] Sidik NP, McEntegart M, Roditi G, Ford TJ, McDermott M, Morrow A (2020). Rationale and design of the British Heart Foundation (BHF) Coronary Microvascular Function and CT Coronary Angiogram (CorCTCA) study. *American Heart Journal*.

[42] Knuuti J, Wijns W, Saraste A, Capodanno D, Barbato E, Funck-Brentano C (2020). 2019 ESC Guidelines for the diagnosis and management of chronic coronary syndromes. *European Heart Journal*.

[43] Genders TSS, Steyerberg EW, Alkadhi H, Leschka S, Desbiolles L, Nieman K (2011). A clinical prediction rule for the diagnosis of coronary artery disease: validation, updating, and extension. *European Heart Journal*.

[44] Rademaker AAEM, Danad I, Groothuis JGJ, Heymans MW, Marcu CB, Knaapen P (2014). Comparison of different cardiac risk scores for coronary artery disease in symptomatic women: do female-specific risk factors matter. *European Journal of Preventive Cardiology*.

[45] Berry C, Morrow AJ, Marzilli M, Pepine CJ (2022). What Is the Role of Assessing Ischemia to Optimize Therapy and Outcomes for Patients with Stable Angina and Non-obstructed Coronary Arteries. *Cardiovascular Drugs and Therapy*.

[46] Rodriguez Lozano PF, Rrapo Kaso E, Bourque JM, Morsy M, Taylor AM, Villines TC (2022). Cardiovascular Imaging for Ischemic Heart Disease in Women: Time for a Paradigm Shift. *JACC: Cardiovascular Imaging*.

[47] Garcia M, Mulvagh SL, Merz CNB, Buring JE, Manson JE (2016). Cardiovascular Disease in Women: Clinical Perspectives. *Circulation Research*.

[48] Aggarwal NR, Patel HN, Mehta LS, Sanghani RM, Lundberg GP, Lewis SJ (2018). Sex Differences in Ischemic Heart Disease: Advances, Obstacles, and Next Steps. *Circulation: Cardiovascular Quality and Outcomes*.

[49] Schmidt KMT, Nan J, Scantlebury DC, Aggarwal NR (2018). Stable Ischemic Heart Disease in Women. *Current Treatment Options in Cardiovascular Medicine*.

[50] Nanna MG, Wang TY, Xiang Q, Goldberg AC, Robinson JG, Roger VL (2019). Sex Differences in the Use of Statins in Community Practice. *Circulation. Cardiovascular Quality and Outcomes*.

[51] Daly C, Clemens F, Lopez Sendon JL, Tavazzi L, Boersma E, Danchin N (2006). Gender differences in the management and clinical outcome of stable angina. *Circulation*.

[52] Humphries KH, Gao M, Pu A, Lichtenstein S, Thompson CR (2007). Significant improvement in short-term mortality in women undergoing coronary artery bypass surgery (1991 to 2004). *Journal of the American College of Cardiology*.

[53] Rahman H, Corcoran D, Aetesam-Ur-Rahman M, Hoole SP, Berry C, Perera D (2019). Diagnosis of patients with angina and non-obstructive coronary disease in the catheter laboratory. *Heart*.

[54] Ford TJ, Stanley B, Sidik N, Good R, Rocchiccioli P, McEntegart M (2020). 1-Year Outcomes of Angina Management Guided by Invasive Coronary Function Testing (CorMicA). *JACC: Cardiovascular Interventions*.

[55] Mygind ND, Michelsen MM, Pena A, Frestad D, Dose N, Aziz A (2016). Coronary Microvascular Function and Cardiovascular Risk Factors in Women With Angina Pectoris and No Obstructive Coronary Artery Disease: The iPOWER Study. *Journal of the American Heart Association*.

[56] Iribarren A, Diniz MA, Merz CNB, Shufelt C, Wei J (2022). Are we any WISER yet? Progress and contemporary need for smart trials to include women in coronary artery disease trials. *Contemporary Clinical Trials*.

[57] Shaw LJ, Pepine CJ, Xie J, Mehta PK, Morris AA, Dickert NW (2017). Quality and Equitable Health Care Gaps for Women: Attributions to Sex Differences in Cardiovascular Medicine. *Journal of the American College of Cardiology*.

[58] Alcalde-Rubio L, Hernández-Aguado I, Parker LA, Bueno-Vergara E, Chilet-Rosell E (2020). Gender disparities in clinical practice: are there any solutions? Scoping review of interventions to overcome or reduce gender bias in clinical practice. *International Journal for Equity in Health*.

[59] Pauly DF, Johnson BD, Anderson RD, Handberg EM, Smith KM, Cooper-DeHoff RM (2011). In women with symptoms of cardiac ischemia, nonobstructive coronary arteries, and microvascular dysfunction, angiotensin-converting enzyme inhibition is associated with improved microvascular function: A double-blind randomized study from the National Heart, Lung and Blood Institute Women’s Ischemia Syndrome Evaluation (WISE). *American Heart Journal*.

[60] Pizzi C, Manfrini O, Fontana F, Bugiardini R (2004). Angiotensin-converting enzyme inhibitors and 3-hydroxy-3-methylglutaryl coenzyme A reductase in cardiac Syndrome X: role of superoxide dismutase activity. *Circulation*.

[61] Handberg EM, Merz CNB, Cooper-Dehoff RM, Wei J, Conlon M, Lo MC (2021). Rationale and design of the Women’s Ischemia Trial to Reduce Events in Nonobstructive CAD (WARRIOR) trial. *American Heart Journal*.

